# Multiple effect of social influence on cooperation in interdependent network games

**DOI:** 10.1038/srep14657

**Published:** 2015-10-01

**Authors:** Luo-Luo Jiang, Wen-Jing Li, Zhen Wang

**Affiliations:** 1 College of Physics and Electronic Information Engineering, Wenzhou University, Wenzhou, 325035, China; 2 Zhejiang DongFang Vocational and Technical College, Wenzhou, 325035, China; 3 Interdisciplinary Graduate School of Engineering Sciences, Kyushu University, Fukuoka 816-8580, Japan; 4 School of Automation, Northwestern Polytechnical University, Xi’an 710072, China

## Abstract

The social influence exists widely in the human society, where individual decision-making process (from congressional election to electronic commerce) may be affected by the attitude and behavior of others belonging to different social networks. Here, we couple the snowdrift (SD) game and the prisoner’s dilemma (PD) game on two interdependent networks, where strategies in both games are associated by social influence to mimick the majority rule. More accurately, individuals’ strategies updating refers to social learning (based on payoff difference) and above-mentioned social influence (related with environment of interdependent group), which is controlled by social influence strength *s*. Setting *s* = 0 decouples the networks and returns the traditional network game; while its increase involves the interactions between networks. By means of numerous Monte Carlo simulations, we find that such a mechanism brings multiple influence to the evolution of cooperation. Small *s* leads to unequal cooperation level in both games, because social learning is still the main updating rule for most players. Though intermediate and large *s* guarantees the synchronized evolution of strategy pairs, cooperation finally dies out and reaches a completely dominance in both cases. Interestingly, these observations are attributed to the expansion of cooperation clusters. Our work may provide a new understanding to the emergence of cooperation in intercorrelated social systems.

Understanding the evolution of cooperation through natural selection is one open challenge in biology science^1^, because altruistic behavior seems inconsistent with Darwinian selection theory^2^. Nevertheless, cooperation is ubiquitous in living organisms, from bacterial colonies to animal and human societies^3^,^4^. Among the existing attempts at constructing a theory of cooperation, evolutionary game theory together with its various extensions has played a central role in addressing the aforementioned problem^5^. A typical, simple example is using the prisoner’s dilemma (PD) game as one paradigm, which has attracted great attention from theoretical and experimental viewpoints^6^. In its basic model, two players are required to make the simultaneous selection from two strategies: cooperation (*C*) and defection (*D*). If both cooperate (defect), they obtain the reward *R* (punishment *P*). If, however, one player cooperates while the other chooses defection, the latter gets the temptation *T* and the former is left with the sucker’s payoff *S*. These payoffs satisfy the ranking *T* > *R* > *P* > *S* and 2*R* > *T* + *S*, from where it is clear that defection maximizes the individual payoff, irrespective of the opponent’s selection. On the other hand, when *T* > *R* > *S* > *P*, the so-called snowdrift (SD) game, as an alternative, appears, where cooperation-defection pairs quickly arise^7^.

To overcome the unfortunate dilemma, a great number of mechanisms have been suggested to sustain cooperation behavior in evolutionary games^2^,^3^. Up to now, the most prominent mechanisms involve kin selection^8^, direct and indirect reciprocity^9^, group selection^10^ and network reciprocity^11^,^12^. Among them, network reciprocity is the most well-known mechanism, which indicates that on the structured populations cooperators can aggregate into compact clusters and so avoid being wiped out by defectors. Although the empirical evidence based on economic experiments indicate that it may be compromised or fail^13^, there still exists an ample place to explore how and why spatial structure promotes cooperation^14^,^15^,^16^, especially with the advent of various complex networks, such as small-world^17^, scale-free^18^, hierarchical^19^ and co-evolving networks^20^,^21^. Recent attention has shifted towards interdependent networks^22^,^23^,^24^, where the slight changes on one network layer may cause catastrophic and very much unexpected consequence on another network^25^. Following this discovery, the robustness of such structure has been tested^26^ and diverse dynamics processes, such as epidemic spreading^27^, diffusion^28^, voting^29^, synchronization^30^ and game model^31^ have been involved as the hot topics (see refs 32,33 for a recent review).

With regard to evolutionary games on interdependent networks, how to construct interdependence between different networks becomes the critical issue. In a recent research work^34^, the biased utility function suppresses the feedback of individual success, which results in a spontaneous separation of time scales on both networks. Consequently, cooperation is enhanced due to deceleration of defection invasion. While in^35^, if the co-evolution between strategy and network structure is established, it is unveiled that the interdependence between both networks can self-organize so as to yield the optimal cooperation level. In spite of great progress with interdependent networks games, vast majority of existing works just consider the simple assumption that different layers have identical social dilemma, namely, the same game in each network layer. While in realistic life, different topology layers usually support diverse actions, which means that various dilemmas may be in presence on different interaction topology. More importantly, another non-negligible fact is that even if the ongoing interactions differ, individual information of strategy choice, to large extent, is affected by the collective selection of other systems rather than completely comes from the local interaction. In this sense, the question, what we will address in what follows, becomes very clear. Namely, if we consider different games on correlated networks and assume individual strategy selection is influence by both internal and external information, is this beneficial for enriching network reciprocity or not?

Aiming to answer the above problem, here we propose the social influence mechanism by assuming that, except for the local interaction, individual may refer to the most popular strategy of the interdependent group on other network with a certain probability *s* (namely, the so-called social influential strength, which exists widely in social systems^36^,^37^,^38^,^39^). It thus becomes to determine how strong such influence strength really oughts to be for the maximal promotion of cooperation on interdependent networks. We show, by means of Monte Carlo simulations, that there is a critical threshold of social influence strength. Below this threshold, it is exhibited that cooperation is separated in both systems and drops to null level with slight increment of social influence strength. However, if the influence strength exceeds the threshold, there is an unexpected, fast outbreak of cooperation behavior, which reaches the completely dominant state (namely, cooperation is promotes best only after an intermediate social influence strength). In the following, we will detail the model and the implementation of the dynamics, present our main results and summarize and discuss them.

## Results

**Figure 1:**
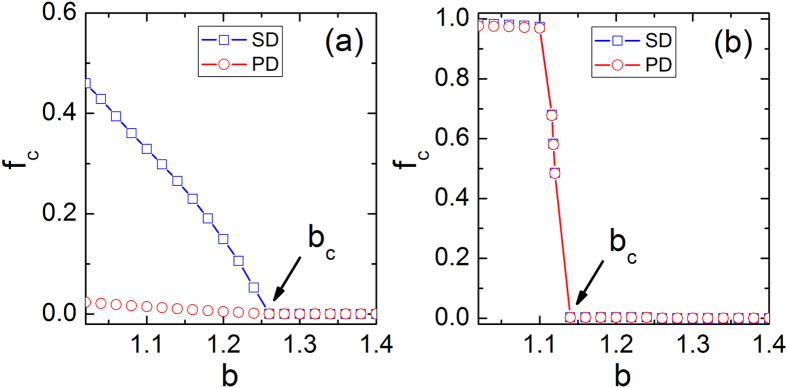
Cooperation frequency *f*_*c*_ as a function of *b* under social influence strength *s* = 0.05 (a) and s = 0.5 (b). Here, SD and PD denote the average value in each network respectively. The black arrows mark the critical points for phase transition.

**Figure 2:**
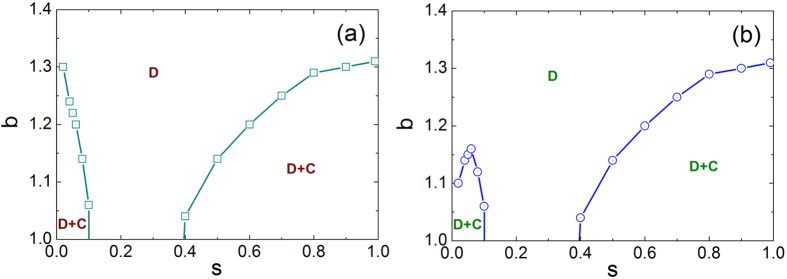
Phase diagrams in *b*-*s* space for SD (a) and PD (b) on each network respectively.

We start by exploring how the social influence drives the evolution of cooperation on interdependent networks. As *s* = 0, our model returns the traditional snowdrift (SD) game and prisoner’s dilemma (PD) game in isolated network. It thus becomes interesting to study the impact of positive *s* on cooperation frequency *f*_*c*_ (see Fig. 1). It is clear that the evolution of cooperation in both systems is completely synchronous at *s* = 0.5, while for *s* = 0.05 such a synchronized tendency is destroyed towards a higher cooperation level in SD, which is similar to the theoretical prediction in single-layer network^4^. Furthermore, there exists the transition from coexisting states of cooperation and defection to full defection state when *b* exceed a critical value *b*_*c*_, marked by black arrows. Along this line, we examine phase diagram between *C* + *D* phase and *C*(*D*) phase in the *b* − *s* space for snowdrift game and prisoner’s dilemma game, respectively. From Fig. 2, one can find that cooperation coexist with defection for both small and large values of *s*. However, intermediate value of *s* induces the extinction of cooperation in both snowdrift game and prisoner’s dilemma game.

**Figure 3:**
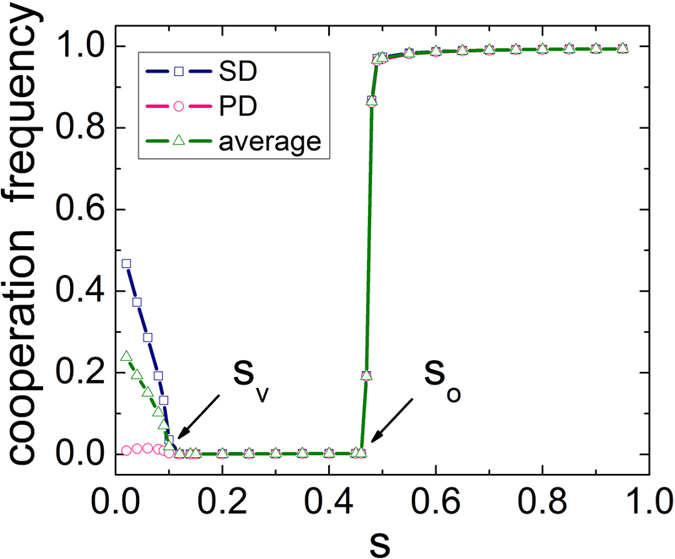
Cooperation frequency as a function of social influence strength *s* on interdependent networks. There are two threshold *s*_*v*_ and *s*_*o*_ for different phase transitions. The used parameter is *b* = 1.1.

To get a clear image about the total impact of influence strength *s*, Fig. 3 features the cooperation frequency *f*_*c*_ in dependence on the influence strength *s* for both games, as well as for the whole system. As evidenced in the figure, we can observe three types of behaviors within the system. When the influence strength *s* is small (which makes interdependent setup close to traditional isolated network), there exists a bias towards the evolution of cooperation, which is similar to the symmetric breaking phenomenon induced by the utility distribution^24^. In this case, cooperation almost vanishes in the prisoner’s dilemma game yet survives in the snowdrift game. This is actually consistent with what we can expect, because cooperation can obtain more benefit when facing a defector in snowdrift game. However, when influence strength exceeds a threshold *s*_*v*_, a completely different scenario appears: cooperation simultaneously dies out in both systems, namely, the increment of social influence is not beneficial for the evolution of cooperation. On the other hand, this observation does not agree with the well-known other-regarding case^40^, which emphasizes the impact of external environment and facilitates cooperation. With further raising *s*, interestingly, we can see that there is an abrupt outbreak of cooperation in both games (at another threshold *s*_*o*_ ≈ 0.47) and cooperation fast reaches an exclusive dominance, which implies that excessive dependence on external environment may bring more benefit for cooperation. Combing with these observations, it is thus suggested that when players refer to external strategy information, multiple effect takes place: increase of social influence strength results in the biased separation, synchronous extinction and abrupt outbreak towards high cooperation frequency. In what follows, we will systematically examine the validity of this claim.

**Figure 4:**
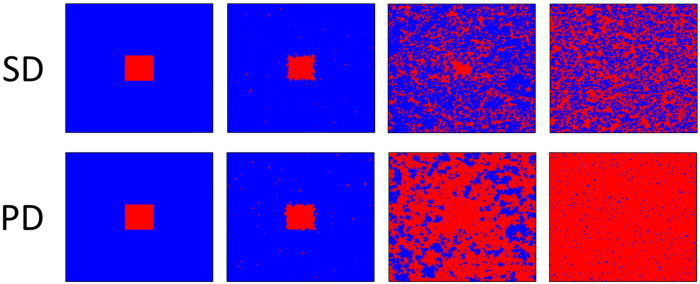
Snapshots of the distribution of cooperators (blue) and defectors (red) on interdependent networks at 1, 10, 50 and 50000 MC steps from left to right. The top (bottom) panel is for SD (PD) game. The used parameters are *s* = 0.05, *b* = 1.1 and *L* = 200.

To understand the impact of social influence more precisely, we can also visually inspect the characteristic spatial evolution of cooperators and defectors for different values of social influence *s*. Figure 4 features snapshots of two interdependent networks at small *s* value, obtained from a prepared initial state (in order to demonstrate the impact of social influence as clear as possible). It is clear that cooperative domain of snowdrift game is able to survive the initial onslaught of defectors and then reaches the steady state fast. At variance, the observation of prisoner’s dilemma game seems completely different: the initial cooperative domain quickly splits into a great deal of smaller clusters, which are further invaded by defectors. In the stationary state (the bottom right panel) defectors dominate, and only a quite small fraction of cooperators is able to survive in the form of isolated clusters. All these observations are actually similar to previous results obtained for a single square lattice^4^, since individual decision-making process just receives feeble influence from external environment.

**Figure 5:**
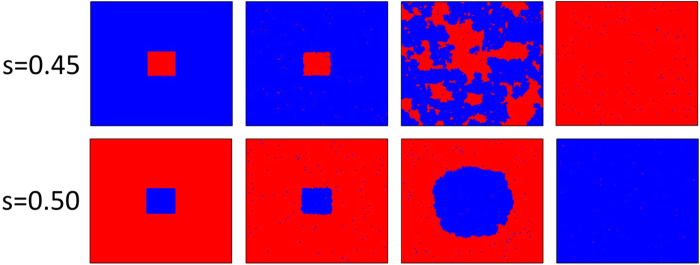
Snapshots of the distribution of cooperators (blue) and defectors (red) for *s* = 0.45 (top) and *s* = 0.5 (bottom) at 1, 20, 2000 and 20000 MC steps from left to right. From Fig. 3, we know both values can guarantee the synchronized evolution, we thus select SD (top) and PD (bottom) for better visual observation of cluster expansion. The used parameters are *b* = 1.1 and *L* = 200.

Interestingly, if we increase influence strength *s*, the above phenomenon will be replaced by the spontaneous emergence of coordination and synchronization of the evolutionary process in both systems. Figure 5 displays the evolution snapshots of cooperator and defectors for two typical influence strength values, below and above the threshold value *s*_*o*_, with similar prepared initial distribution of Fig. 4. It is obvious that, irrespective of which influence strength, cooperation domains synchronously split, vanish or expand in both networks, as the previous report of utility function involving external players^41^. For middle *s*, since cooperator clusters can not get enough powerful support from the external environment, which naturally resorts to the undisputed dominance of defectors^42^. However, when we increase the social influence, the most visible change takes place: in the very early stages of games defectors are able to plunder very effectively, which results in the remaining cooperators to form small clusters. Then, these clusters will quickly expand and finally merge into one giant domain (see bottom panel of Fig. 5), which has completely identical location in both networks (namely, spontaneous emergence of coordination and synchronization of evolution process). As predicted in the case of joint utility function on interdependent networks^41^, this trend of synchronization generates the interdependent network reciprocity and provides beneficial condition for the final dominance of cooperation. Thus, whether there exists the coordination of sufficient strong cooperation domains on both networks is crucial for the existence and expansion of cooperation.

**Figure 6:**
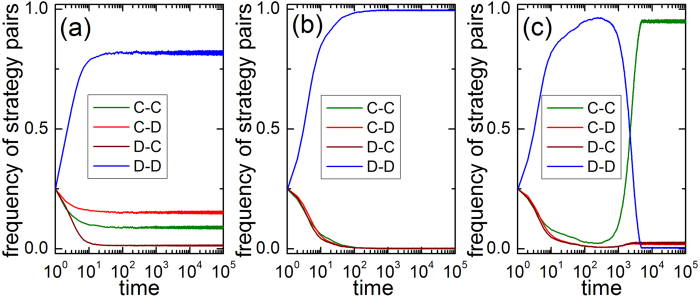
Time course of frequency of strategy pairs for (a) *s* = 0.05, (b) *s* = 0.45 and (c) *s* = 0.5. *C* − *C* pair indicates the connection between two cooperators in the whole system, and *D* − *D* pair denotes the connection between two defectors in the whole system. While *C* − *D* indicates connection between cooperators and defectors only in SD, *D* − *C* pair denotes connection between cooperators and defectors only in PD. The used parameters are *b* = 1.1.

Next, it is interesting to elucidate why social influence induces multiple effect on the evolution of cooperation. In Fig. 6, we inspect the time courses of strategy pairs between networks for different values of influence strength. *C* − *C* pair indicates the connection between two cooperators in the whole system, and *D* − *D* pair denotes the connection between two defectors in the whole system. While *C* − *D* indicate connection between cooperators and defectors only in SD, *D* − *C* pair denotes connection between cooperators and defectors only in PD. Starting from random distribution, the value of *C* − *D* pair is always higher than *D* − *C* pair, which explains why cooperation frequency in SD for small *s* (*s* = 0.05). However, as *s* increases, the evolution of heterogeneous strategy pairs (namely, *C* − *D* and *D* − *C*) become synchronized and goes to extinction together. For intermediate value of *s* (*s* = 0.45), the frequency of *D* − *D* pair fast reaches stable state and completely dominates system. At variance, large *s* enables the frequency of *D* − *D* firstly reach a meta-stable state. But due to effective expansion of the remaining cooperation clusters, *C* − *C* pair fast replaces *D* − *D* and holds the dominance in the final state. These results are consistent with the evolution of snapshots in Figs 4 and 5.

**Figure 7:**
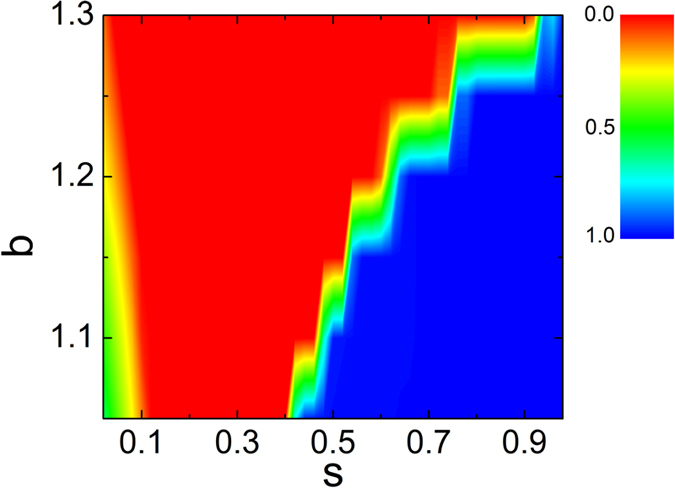
Average cooperation frequency in *b*-*s* space. The color represents cooperation frequency.

To give a holistic profile, the multiple effect of social influence on average cooperation frequency in *b* − *s* space is in Fig. 7. For small value of social influence *s*, there still exist a few cooperators in the system; while these cooperators vanish when *s* increases because intermediate social influence damages formation of cooperation clusters. It is interesting that the cooperation is greatly maintained for large *s*. These seem to indicate that the promotion effects only works when competition between social influence and social learning leads to the synchronized growth of cooperation clusters, as reported in ref. 43, where conformity driven by the social influence favors cooperation.

## Discussion

To conclude, we have studied the evolution of cooperation in prisoner’s dilemma game and snowdrift game on interdependent networks that are subject to interconnectedness by means of the so-called “social influence” mechanism^44^,^45^,^46^. Except for imitating the strategy of local neighbor, player is also allowed to learn the most popular strategy from other network based on the influence strength *s*. By means of systematic simulations, we have shown that such a mechanism can lead to the multiple effect on the evolution of cooperation, which is different from games played in isolated network^47^. Small social influence causes the asymmetric cooperation, which is higher in snowdrift game (similar to traditional setup). Large social influence enhances the tendency to cooperate, resulting from the fast and synchronized expansion of cooperative clusters on different networks. However, the intermediate social influence strength inhibits cooperation. Our model not only incorporates social influence into cooperative dynamics^48^, but also involves the information exchange of different games in multilayer framework^49^. It thus may open a new door to understand the controllability of cooperation via social influence.

## Methods

We consider two different evolutionary games, namely the prisoner’s dilemma (PD) and the snowdrift (SD) games, on two regular, *L* × *L* square lattices with nearest-neighbors interactions and periodic boundary conditions. More specifically, one network is used to support prisoner’s dilemma game, while snowdrift game is carried out on the other network. The payoffs in both games follow the standard procedure to govern the social dilemma. The prisoner’s dilemma game is characterized by the temptation to defect *T* = *b*, reward for mutual cooperation *R* = 1, and punishment *P* = 0.2 as well as the sucker’s payoff *S* equaling 0, whereby 1 < *b* ≤ 2 ensures a proper payoff ranking^4^. It is worth mentioning that qualitatively similar results are obtained also for negative values of *S*. While for snowdrift game, we just need to take a negative value of *P* to keep the payoff ranking. For simplicity (yet without loss of generality), we set *P* = −0.2 throughout this paper.

Regardless of the type of the game, initially each individual on site *x* of both networks is designated either as a cooperator (*s*_*x*_ = C) or defector (*s*_*x*_ = D) with equal probability. Then, every player has one partner located in the same position of another network for possible information sharing (*i.e.*, point-to-point interdependency as the generalization setup^24^). The evolutionary process proceeds via the Monte Carlo simulation procedure, comprising the following elementary steps. First, a randomly selected player *x* of random chosen network acquires its accumulate payoff *P*_*x*_ by playing the games with all its neighbors. Next, player *x* updates its strategy according to the collective strategy information of another network or the payoff information of local topology, which is detailed as follows.

- We incorporate “social influence” into interdependent network games, which means that the individual behavior choice is affected by the state of corresponding group (consisting of parter 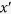 and its four neighbors) on the other network. Under this scenario, player *x* learns the most popular strategy of that group with the social influential strength *s* (0 ≤ *s* ≤ 1). To consider the influence of noise, it is allowed that player randomly choose one strategy with probability 0.1. It is worth mentioning that the size of such a group may play a significant role in determining the final results, as reported in^50^.
- On the contrary, if the above “social influence” event does not take place, player resorts to the classical updating fashion as the case of single network^4^. Player *x* chooses one of its nearest neighbors at random, and the chosen player *y* also acquires its payoff *P*_*y*_ in the same way as player *x*. Subsequently, player *x* attempts to adopt the strategy *s*_*y*_ from player *y* with a probability determined by the Fermi function

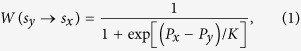


where *K* = 0.1 quantifies the uncertainty related to the strategy adoption process^3^,^4^, which is usually associated with errors in decision making and imperfect information transfer between the players. During a full Monte Carlo (MC) step, each player on both networks is selected once on average to change its strategy.

Importantly, different from existing achievements where the interdependence affects the utility of players^22^,^24^,^34^,^35^, this novel framework allows strategies to be transferred or mimicked across two networks. The parameter 0 ≤ *s* ≤ 1 determines the strength of social influence. Obviously, *s* equaling to 0 returns the case of traditional single network^4^, which means both networks are decoupled. While its increment means that more potential influence of individual strategy choice comes form external environment.

Presented results were obtained by means of Monte Carlo simulations on lattices of linear size varying from *L* = 200 to 500 in order to avoid finite size effects. The necessary relaxation times varied between 10^5^–10^7^ MC steps. Moreover, since the initial distribution of strategies may involve additional disturbances, final results were averaged over up to 30 independent runs to further improve accuracy.

## Additional Information

**How to cite this article**: Jiang, L.-L. *et al.* Multiple effect of social influence on cooperation in interdependent network games. *Sci. Rep.* **5**, 14657; doi: 10.1038/srep14657 (2015).
